# Seizure activity results in calcium- and mitochondria-independent ROS production via NADPH and xanthine oxidase activation

**DOI:** 10.1038/cddis.2014.390

**Published:** 2014-10-02

**Authors:** S Kovac, A-M Domijan, M C Walker, A Y Abramov

**Affiliations:** 1 UCL Institute of Neurology, University College London, Queen Square, London WC1N 3BG, UK; 2 Department of Neurology, University of Muenster, Muenster 48149, Germany; 3 Faculty of Pharmacy and Biochemistry, University of Zagreb, Zagreb 10000, Croatia

## Abstract

Seizure activity has been proposed to result in the generation of reactive oxygen species (ROS), which then contribute to seizure-induced neuronal damage and eventually cell death. Although the mechanisms of seizure-induced ROS generation are unclear, mitochondria and cellular calcium overload have been proposed to have a crucial role. We aim to determine the sources of seizure-induced ROS and their contribution to seizure-induced cell death. Using live cell imaging techniques in glioneuronal cultures, we show that prolonged seizure-like activity increases ROS production in an NMDA receptor-dependent manner. Unexpectedly, however, mitochondria did not contribute to ROS production during seizure-like activity. ROS were generated primarily by NADPH oxidase and later by xanthine oxidase (XO) activity in a calcium-independent manner. This calcium-independent neuronal ROS production was accompanied by an increase in intracellular [Na^+^] through NMDA receptor activation. Inhibition of NADPH or XO markedly reduced seizure-like activity-induced neuronal apoptosis. These findings demonstrate a critical role for ROS in seizure-induced neuronal cell death and identify novel therapeutic targets.

Reactive oxygen species (ROS) contribute to neuronal damage and have been linked to excitotoxicity.^1, 2, 3, 4^ An increase in ROS generation has also been identified in acute neurologic disease such as stroke,^5,6^ and recent evidence indicates that this may contribute to neuronal damage in seizures and epilepsy.^7, 8, 9, 10^ However, ROS measurements during seizure-like activity were predominantly performed in homogenates, extracellular fluids or brain regions with no clear demonstration of whether the ROS were of neuronal origin.^9,11,12^ Moreover, these studies lacked the necessary temporal resolution to determine accurately the evolution of ROS generation during and after prolonged seizure activity. Such obstacles can be overcome by live cell imaging of ROS, which has emerged as a powerful tool to study disease mechanisms.^13^

If seizure activity induces ROS production in neurons, then a critical question is which sources of ROS production are triggered by such activity. Previous studies have suggested that mitochondria are the primary source of ROS generation in seizure models.^8,14^ However, there are alternative sources of ROS, in particular the enzymes NADPH oxidase and xanthine oxidase (XO). How these contribute to excitotoxicity during seizure activity is uncertain. That these enzymes may have an important role in seizure-induced ROS generation is suggested by two observations: (1) NMDA receptors have a pivotal role in seizure-induced neuronal damage^15^ and (2) direct pharmacologic activation of NMDA receptors can activate NADPH oxidase, increasing free radical production and consequently neuronal death.^5,16,17^ There is also burgeoning evidence of a role for NADPH oxidase activation in chronic brain pathology secondary to psychosocial stress, which leads to the development of neuropathologic alterations, and also in neurodegenerative disease.^18,19^

Acute activation of NADPH oxidase in neurons has mainly been shown after direct pharmacologic activation of NMDA receptors via exposure to high levels of NMDA and this activation is calcium-dependent.^16,17^ More recently, activation of NADPH oxidase has been shown during seizure activity.^9,20^ These pathways also involved NMDA receptor activation and upregulation of NMDA receptor subunits NR1 and NR2B. Nonetheless, these studies used chemoconvulsant epilepsy models, which, in themselves, may have an impact on ROS generation. The mechanisms and relevance of activation of NADPH oxidase during seizure activity independent of chemoconvulsants is unclear, especially given the presence of alternative sources of ROS production. Moreover, XO may also represent a major potential source of ROS during periods of increased metabolism, such as that occuring during seizures. We have therefore asked whether NMDA receptor activation has a role in seizure-induced ROS production and which sources and mechanisms of ROS production are involved in its time course during seizure-like activity.

Here, we demonstrate increased ROS generation during seizure-like activity. This is activity-dependent, but it is maintained by a Ca^2+^-independent pathway involving the activation of NMDA receptors, NADPH oxidase and XO at a later phase. Blocking NADPH oxidase and XO prevented seizure-induced neuronal cell death *in vitro*. We thus provide compelling evidence that these ROS-generating pathways are appropriate targets for preventing neuronal death in seizures.

## Results

### ROS generation and lipid peroxidation *in vitro* in the low magnesium model

**Figure 1:**
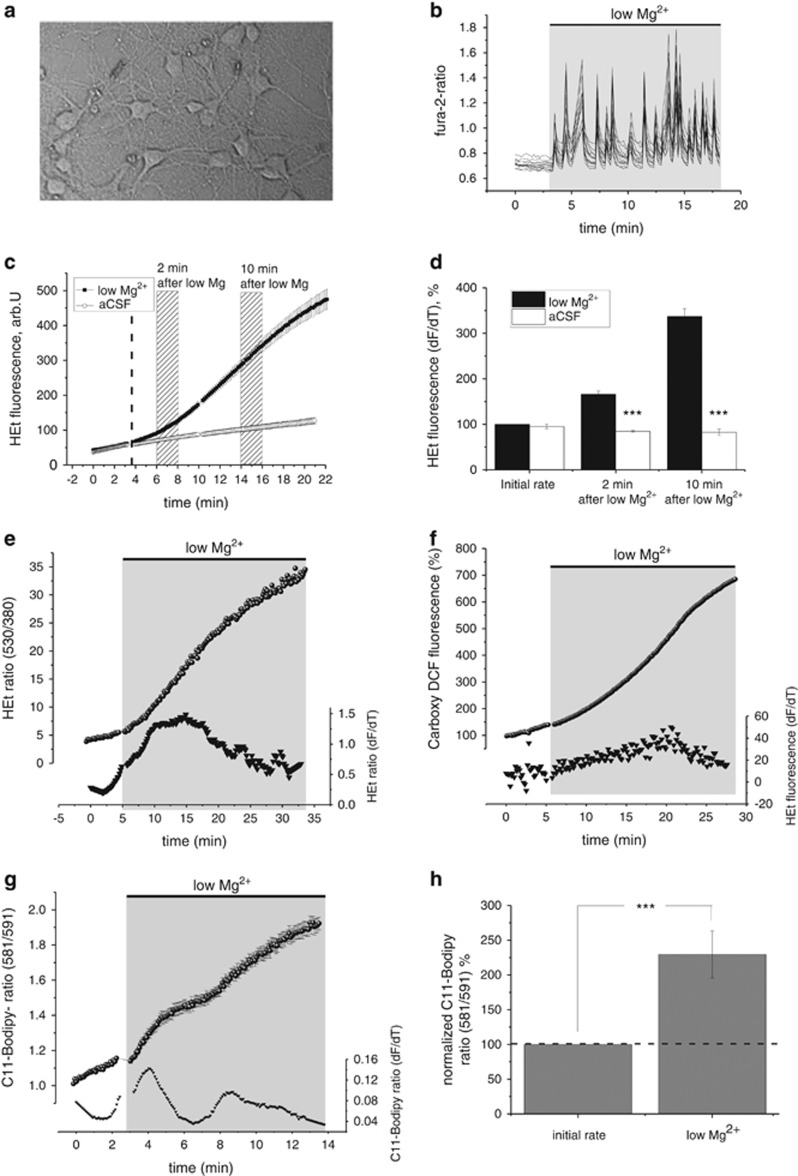
Seizure-like activity in the low magnesium model of epilepsy induces ROS production in primary cortical neurons. (**a**) Phase-contrast image showing rodent glioneuronal cultures *in vitro* and (**b**) seizure-like activity induced in neurons in the low magnesium model, as indicated by repetitive Ca^2+^ oscillations measured with the calcium indicator fura-2 (see also Supplementary Video 1). Each trace in (**b**) represents a single neuron. Note that Ca^2+^ transients in neurons are synchronous and rhythmic characteristic of seizure-like activity. (**c**) HEt fluorescence measurements in neurons (mean±S.E.M.) from a representative experiment showing low-magnesium-induced increases in the HEt fluorescence in neurons exposed to low magnesium treatment (solid circle) and stable rates of HEt fluorescence indicating stable ROS generation rates in neurons treated with aCSF only (control; unfilled circle). The vertical dashed line indicates replacement of the solution to either low magnesium (solid circle) or aCSF (unfilled circle) after a baseline with aCSF. Bars indicate representatives periods that were taken to analyse the rate of ROS generation. (**d**) Mean rates of ROS generation at the representative time points in neurons undergoing low magnesium treatment and neurons treated with aCSF only with the basal rate in neurons set as 100%. ROS generation in neurons as measured with (**e**) ratiometric Het fluorescence imaging and (**f**) using H_2_DFFDA. Traces in (**e**) and (**f**) show fluorescence measurements from single representative neurons (circles). Bottom traces (triangles) in these two panels indicate the differentiated trace. (**g**) Lipid peroxidation in neurons during low-magnesium-induced seizure-like activity as measured with C11-BODIPY ratio in a representative experiment (mean±S.E.M.; upper trace). The bottom traces (triangles) indicates the differentiated mean C11-BODIPY fluorescence of the trace represented above. (**h**) Histogram summarizing rates of lipid peroxidation during baseline (initial rate) and during low magnesium seizure-like activity (low Mg^2+^). Error bars indicate S.E.M. ***P<0.001 and **P<0.01

To determine the mechanisms and sources of ROS generation, and their exact temporal relationship during the initial phase of network hyperexcitability, the well-established low magnesium culture model of seizure-like activity was used.^21,22^ We performed live cell imaging experiments in rat neocortical glioneuronal cocultures (days *in vitro* 12–21; Figure 1a). In keeping with previous reports, we found that seizure-like activity in the low magnesium model induced oscillatory increases in intracellular Ca^2+^, monitored with fura-2 (Figure 1b and Supplementary Video 1),^23^ coinciding with rapid burst firing of neurons, as has been shown previously.^24^ These changes resulted in an immediate and significant increase in the rate of ROS production in neurons (Figure 1c), while the rate of ROS production in control cultures (neurons treated with aCSF) was no different from baseline (Figure 1c). The response was not homogeneous in all cells. Based on a qualitative analysis of the rates of dihydroethidium (HEt) fluorescence, we could distinguish two phases: ~2 min and 10 min after low magnesium exposure, and these time windows were thus used for analysis (Figure 1c). There was a statistically significant difference of ROS production between the low magnesium and control (repeated-measures ANOVA; F (1, 223)=56.4; *P*<0.001). There was also a significant interaction between time and group (F (1,223)=26.4, *P*<0.001), indicating that as time progressed the difference between the groups became significantly larger (Figure 1d). Rates of ROS production measured after 2 min in neurons exposed to low magnesium (*n*=188) were almost two times that of control neurons (*n*=41; 166±7% *versus* 85±2%). There was a further increase in the rate of ROS production in neurons observed 10 min after omission of magnesium; ROS production in these neurons was almost fourfold greater than that seen in neurons under control conditions (*n*=125; 337±17% *versus* 83±7%). To confirm ROS production using different approaches or indicators, we conducted identical experiments and obtained similar results using 530/380 ratio or measurements of 5- and 6- carboxy-2',7'-dichlorofluorescein (H_2_DFFDA) fluorescence (Figures 1e and f).

ROS are known to induce oxidative degradation of lipids (lipid peroxidation). We therefore determined lipid peroxidation in the low magnesium culture model of seizure-like activity using C11-BODIPY, an indicator of lipid peroxidation. The rate of lipid peroxidation increased ~2-fold as a result of low-magnesium-induced seizure-like activity (*n*=32; *P*<0.001; paired *t*-test; Figures 1g and h).

Omission of magnesium from the superfusion solution induces oscillatory increases in neuronal [Ca^2+^]_c_ (Kovacs *et al.*;^25^ Figure 1b). We hypothesized that increases in neuronal ROS reflect the activity status of neurons. Simultaneous [Ca^2+^]_c_ and ROS measurements indicated that the neuronal ROS production coincided with the Ca^2+^ signal and was greater in neurons with larger and more sustained Ca^2+^ oscillations (Supplementary Figures 1A and B).

**Figure 2:**
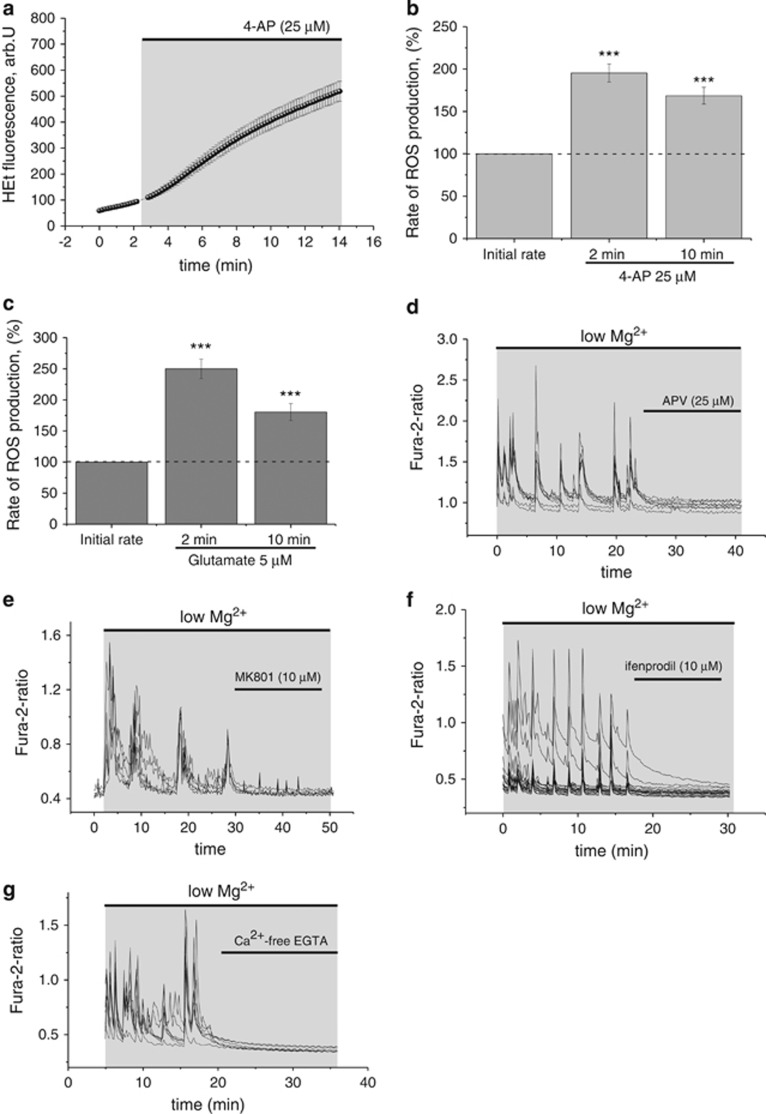
ROS changes in neurons during 4-AP and glutamate-induced epileptiform activity. (**a**) HEt fluorescence measurements in neurons (mean±S.E.M.) from a representative experiment showing 4-AP induced increases in the HEt fluorescence in neurons (solid circle). (**b**+**c**) Histogram summarizing ROS changes in neurons in (**b**) the 4-AP model and in (**c**) glutamate model of seizure-like activity (mean±S.E.M.). ***P<0.001. NMDA receptor sensitivity of low-magnesium-induced Ca^2+^ signals in neurons. Blocking the NMDA receptor with (**d**) APV (25 *μ*M), (**e**) MK801 (10 *μ*M), (**f**) ifenprodil (10 *μ*M), an antagonist of NR2B subunit of NMDA receptor, or (**g**) omitting Ca^2+^ and adding the Ca^2+^ chelator (+EGTA 0.5 mM) to the external solution abolished the established low-magnesium-induced Ca^2+^ signal in neurons. Low-magnesium-induced Ca^2+^ signals in neurons are presented as fura-2 ratios. Each trace represents a single neuron

To confirm that increases in ROS production were not a peculiarity of the low magnesium model of seizure-like activity, we reproduced our findings using another *in vitro* epilepsy model. A well-established convulsant,^23,26,27^ 4-aminopyridine (4-AP), also induced a ~1.5- to 2-fold increase in ROS production compared with baseline at both time points (*n*=126; 2 min: 195±11% 10 min: 168.5±10% repeat-measures ANOVA; F (1, 125)=464.6; *P*<0.001; Figures 2a and b), confirming that the increase in ROS production is not model-specific. In addition, we measured ROS production after activation with low concentrations of glutamate, which has been used as an *in vitro* model of epilepsy,^28^ yielding similar results (*n*=76; 2 min: 262±18% 10 min: 179±12% (glutamate); repeat-measures ANOVA; F (1, 75)=291.0; *P*<0.001; Figure 2c).

Unfortunately, we could not measure ROS with genetically encoded ROS probes such as HyPer-3, as treatment with low magnesium induced acidification; this was shown with the H_2_O_2_-insensitive version of HyPer-3, HyPer-C199S (*n*=6; Supplementary Figure 2E). Changes in pH were confirmed using SNARF, which also demonstrated neuronal acidification during low-magnesium-induced seizure-like activity (*n*=29; Supplementary Figures 2A–D).

### Contribution of NMDA receptors and extracellular Ca^2+^ to low-magnesium-induced ROS generation in neurons *in vitro*

Low-magnesium-induced Ca^2+^ oscillations, representing seizure-like activity, have been shown to be NMDA receptor-sensitive.^29^ Blocking the NMDA receptor with NMDA receptor antagonists (APV: 25 *μ*M; *n*=30 or MK801: 10 *μ*M; *n*=56) or the NR2B subunit specific antagonist ifenprodil (10 *μ*M; *n*=73) abolished the low-magnesium-induced Ca^2+^ signal (Figures 2d–f). Ca^2+^-free low magnesium solution also abolished the low-magnesium-induced Ca^2+^ signal, suggesting that external calcium is pivotal in maintaining low-magnesium-induced Ca^2+^ oscillations in neurons (*n*=37; Figure 2g).

**Figure 3:**
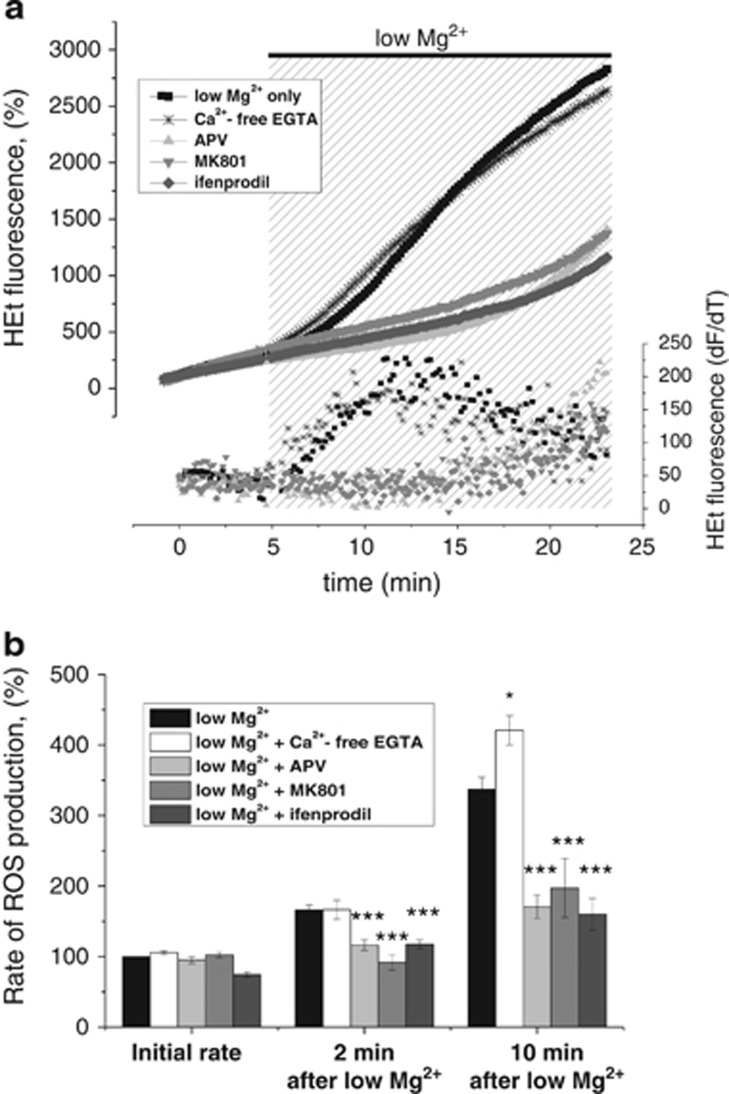
Ca^2+^ and NMDA receptor involvement in low-magnesium-induced ROS generation in neurons. (**a**) The upper panel represents HEt fluorescence in single, representative neuron during low magnesium seizure-like activity, whereas scatter plot in the bottom panel represents the differentiated signal as shown in the upper panel reflecting the rate of ROS generation. Antagonizing NMDA receptors with APV (25 *μ*M), MK801 (10 *μ*M) or with antagonist of the NRB2 subunit of NMDA receptor ifenprodil (10 *μ*M) abolished the low-magnesium-induced increase in ROS generation. Note that neuronal ROS generation was not abolished in low Mg^2+^ Ca^2+^-free aCSF. (**b**) Histogram summarizing mean±S.E.M. rates of ROS generation at 2 and 10 min after omission of magnesium from the extracellular solution for the different treatment groups. *P<0.05 and ***P<0.001

Given that the [Ca^2+^]_c_ oscillations in the low magnesium model are dependent on NMDA receptors and external Ca^2+^ entry, we determined how each of these factors contributed to ROS generation observed in this model. We measured HEt fluorescence in the presence of different NMDA receptor blockers (MK801, APV and ifenprodil) or Ca^2+^-free low magnesium aCSF and compared rates of ROS production with the rates obtained with low magnesium treatment alone (Figures 3a and b).

There was a statistically significant difference in the rate of ROS production between the groups (repeat-measures ANOVA; F (4, 405)=19.5; *P*<0.001). There was also a significant interaction between time and group F (4, 405)=11.8, *P*<0.001), indicating that as time progressed the difference between the groups became significantly larger (Figure 3b). Blocking the NMDA receptor in neurons with APV (*n*=56; 25 *μ*M), MK801 (*n*=65; 10 *μ*M) and ifenprodil (*n*=52; 10 *μ*M) in low magnesium conditions significantly reduced the rate of ROS production both 2 min and 10 min after omission of magnesium when compared with control (Figures 3a and b). Despite blocking low-magnesium-induced [Ca^2+^]_c_ signals in neurons (Figure 2g), Ca^2+^-free low magnesium aCSF had no suppressive effect on neuronal low-magnesium-induced ROS formation (Figures 3a and b). Neuronal rates of ROS generation in Ca^2+^-free low magnesium did not differ significantly from neuronal rates of ROS generation in low magnesium conditions at 2 min (Figure 3b) and ROS production at 10 min was even slightly higher in neurons exposed to Ca^2+^-free low magnesium compared with low magnesium only, indicating, surprisingly, that seizure-induced ROS generation is not dependent on Ca^2+^ influx.

As ROS generation is NMDA receptor dependent when Ca^2+^ is present, we hypothesized that ROS generation in Ca^2+^-free low magnesium aCSF would also depend on NMDA receptor activation.

**Figure 4:**
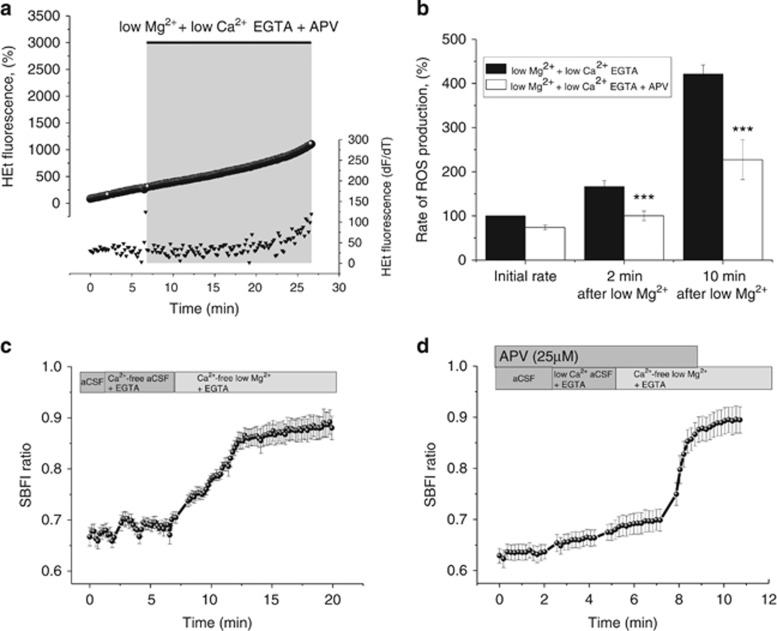
NMDA receptor and sodium current involvement in low magnesium Ca^2+^-free EGTA induced ROS generation. (**a**) HEt fluorescence from a representative neuron under low Mg^2+^- and Ca^2+^-free conditions and NMDA receptor antagonism with APV (25 *μ*M; circles). The bottom trace (triangles) shows the HEt signal after differentiation (triangles) reflecting the rate of ROS generation. Note that ROS generation is abolished under these conditions. (**b**) Histogram summarizing the mean rates of ROS generation in neurons under low Mg^2+^- and Ca^2+^-free conditions (black) compared with low Mg^2+^- and Ca^2+^-free conditions in the presence of APV (25 *μ*M; white). (**c**) Intracellular sodium levels in neurons as measured with SBFI increased after omission of Mg^2+^- from Ca^2+^-free aCSF. (**d**) This increase can be blocked with the NMDA receptor antagonist APV (25 *μ*M). Traces in (**c**) and (**d**) show the mean±S.E.M. value of the SBFI fluorescent signal measured in all neurons in one representative experiment. ***P<0.001

There was a statistically significant difference of ROS production between neurons treated with APV (25 *μ*M; *n*=25) in Ca^2+^-free low magnesium aCSF and neurons treated with Ca^2+^-free low magnesium aCSF, only (repeat-measures ANOVA; F (1, 111)=24.0; *P*<0.001; Figures 4a and b). There was a statistically significant interaction between the effects of group (APV+Ca^2+^-free low magnesium aCSF *versus* Ca^2+^-free low magnesium aCSF) and time point measured on ROS production, indicating that as time progressed the difference between the groups became significantly larger (F (1, 111)=6.0; *P*<0.05). Blocking NMDA receptors with APV (25 *μ*M; *n*=25) significantly reduced the ROS increase in Ca^2+^-free low magnesium aCSF both 2 and 10 min after low magnesium treatment (Figures 4a and b). Imaging of intracellular sodium with SBFI showed that omission of magnesium from Ca^2+^-free aCSF induced an immediate rise of intracellular sodium (*n*=87; Figure 4c). This intracellular sodium rise was blocked with APV (*n*=32; Figure 4d), indicating that ROS production in Ca^2+^-free low magnesium conditions can be maintained by a current through the NMDA receptor linked to an increase in intracellular sodium through NMDA receptors (Figures 4c and d).

### Sources of ROS generation in low magnesium model

**Figure 5:**
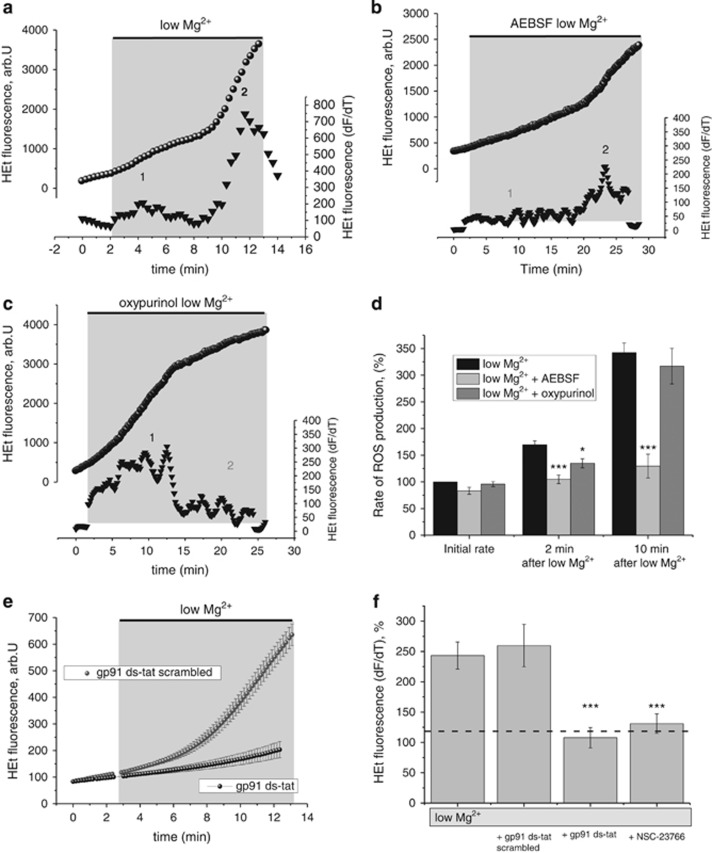
Two distinct phases of ROS generation were seen in the low magnesium model of epilepsy: (**a**) Two distinct phases of ROS generation were observed after neuronal exposure to low magnesium condition. The bottom trace represents the differentiation of the signal (triangle) and thus reflects the rate of ROS generation in the neuron. The numbers (1 and 2) highlight the key phases of ROS generation in the bottom traces showing the differentiated HEt signal. The key mechanisms of ROS generation were identified by (**b**) antagonism of the NADPH oxidase with AEBSF (20 *μ*M), which abolished phase 1, and (**c**) XO inhibition with oxypurinol (20 *μ*M), which abolished phase 2. Traces in (**a**–**c**) show fluorescence measurements from single neurons (circles). (**d**) Histogram summarizing the impact of NADPH oxidase and XO inhibitors on low-magnesium-induced the ROS generation at different time points. Error bars indicate S.E.M. ***P<0.001 and * *P*<0.05. Effect of NADPH oxidase inhibitor gp91 ds-tat and Rac1 activator NSC-23766 on ROS production in the low magnesium model of seizure-like activity: (**e**) Het fluorescence measurements in the low magnesium model after blocking with gp91 ds-tat, an NADPH oxidase assembly inhibitor. Each trace represents representative experiments (mean±S.E.M.); neurons pretreated with gp91 ds-tat (black trace) and neurons pretreated with scrambled gp91 ds-tat (grey trace). (**f**) Histogram summarizing the effect of gp91 ds-tat scrambled, NADPH oxidase assembly inhibitor gp91 ds-tat and inhibitor of Rac1 activator NSC-23766 on ROS changes during seizure-like activity. Error bars indicate S.E.M. ***P<0.001

Recent studies have suggested that excessive pharmacologic NMDA receptor activation predominantly induces ROS production via activation of NADPH oxidase.^17^ Given that low-magnesium-induced ROS generation was NMDA receptor dependent, we asked whether ROS generation can be blocked by inhibiting NADPH oxidase with AEBSF (20 *μ*M), a well-established inhibitor of NADPH oxidase activation.^30^ Excessive neuronal activity has been linked to ATP depletion.^23^ Intracellular ATP depletion results in increased adenine formation and consequently an increase in hypoxanthine and xanthine, substrates for XO.^31,32^ We therefore hypothesized that late phases of ROS generation in the low magnesium model might be due to hypoxanthine and xanthine oxidation by XO, a process that generates hydrogen peroxide. To distinguish between these two pathways, we blocked low-magnesium-induced ROS production using the NADPH oxidase blocker AEBSF or the XO blocker oxypurinol. Both AEBSF and oxypurinol reduced ROS production (Figures 5a–d). There was a statistically significant difference in the rate of ROS production between the groups (repeat-measures ANOVA; F (1, 292)=14.2; *P*<0.001; Figure 5d). There was a significant interaction between time and group as time progressed; the difference between the groups became significantly larger (F (1, 292)=8.7; *P*<0.001; Figure 5d). ROS production at 2 and at 10 min was significantly lower if AEBSF (*n*=41; 20 *μ*M) was added to low magnesium aCSF when compared with low-magnesium-treated neurons only (Figures 5a, b and d). Inhibition of XO with oxypurinol (*n*=34; 20 *μ*M) abolished the late phase of low-magnesium-induced ROS production (>15 min after oxypurinol application; Figure 5c). These cells showed a high rate of ROS generation in the early phase (Figures 5c and d). In addition, 30 min preincubation with gp91 ds-tat (*n*=53), a specific peptide inhibitor of NADPH oxidase assembly,^33^ inhibited ROS production in the low magnesium model of seizure-like activity when compared with gp91 ds-tat scrambled (*n*=43; *P*<0.001; *t*-test; Figures 5e and f). We next blocked Rac1, an activator of the NOX complex.^34^ Inhibition of Rac1 with NSC-23766 (*n*=73) prevented seizure-induced ROS increase when compared with low magnesium treatment only (*n*=54; *P*<0.001; *t*-test; Figure 5f).

We failed to capture the secondary increase in ROS and its inhibition with XO by statistical analysis owing to the time differences in the appearance of the secondary ROS generation increase as can be seen by qualitative visual assessment of the raw data (Supplementary Figure 3). Unlike oxygen deprivation,^5^ seizure-like activity is a more subtle and variable dysfunction with neuronal excitotoxicity and cell death occurring at different time points.

**Figure 6:**
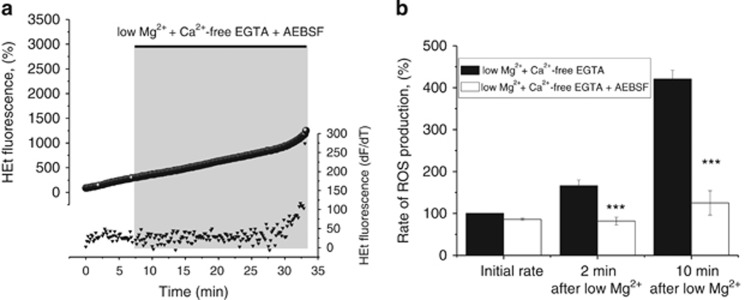
NADPH oxidase is the main source of ROS generation in neurons exposed to low magnesium Ca^2+^-free EGTA: (**a**) HEt fluorescence measurements from a representative neuron under low Mg^2+^- and Ca^2+^-free conditions when blocking NADPH oxidase with AEBSF (20 *μ*M; circles); bottom trace (triangles) indicates the trace after differentiation. (**b**) Histogram showing the mean ROS generation in neurons under low Mg^2+^- and Ca^2+^-free conditions with (empty bars) and without AEBSF (black bars) at different time points. Error bars indicate S.E.M. ***P<0.001

There was a statistically significant difference in the rate of ROS production between neurons treated with AEBSF (20 *μ*M) in Ca^2+^-free low magnesium aCSF and neurons treated with Ca^2+^-free low magnesium aCSF, only (repeat-measures ANOVA; F (1, 122) =75.1; *P*<0.001). There was a significant interaction between time and group, as time progressed the difference between the groups became significantly larger (F (1, 122) =24.5; *P*<0.001; Figures 6a and b). Blocking NADPH oxidase with AEBSF (20 *μ*M) significantly reduced rates of ROS generation 2 and 10 min after superfusion of low magnesium Ca^2+^-free aCSF when compared with low magnesium Ca^2+^-free treatment only (*n*=36; Figures 6a and b). Similarly, AEBSF blocked ROS production in 4-AP and in response to glutamate (*n*=126, 195±11% (4-AP) *versus n*=82 99±9% (4-AP and AEBSF) and *n*= 76; 262±18 (glutamate) *versus n*=70 151±13% (AEBSF and glutamate); both *P*<0.001; *t*-test; Supplementary Figure 4).

**Figure 7:**
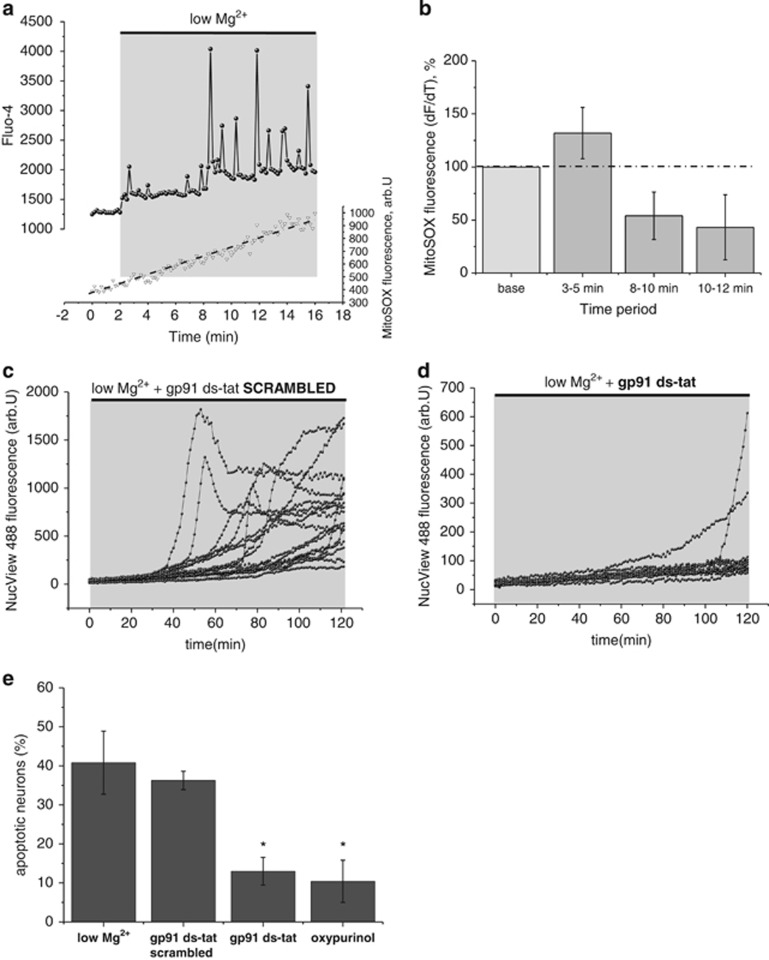
ROS of mitochondrial origin/NADPH oxidase and XO inhibition and apoptosis *in vitro*: (**a**) The upper trace shows a representative low-magnesium-induced [Ca^2+^]_c_ signal recorded using Fluo-4. The bottom trace shows the MitoSOX signal that was recorded simultaneously. (**b**) Histogram summarizing mean±S.E.M. rates of mitochondrial free radical generation in neurons at different time points. (**c**+**d**) Real-time measurements of apoptosis with NucView 488. An increase in NucView 488 fluorescence indicates activation of caspase-3 and thus apoptosis. Each trace represents a single neuron. Note that within the low magnesium aCSF and (**c**) scrambled gp91 ds-tat peptide treatment group, first neurons undergo apoptosis after ~40 min, whereas (**d**) inhibition of NADPH oxidase with gp91 ds-tat delays time to apoptosis. (**e**) Histogram summarizing mean±S.E.M. percentage of apoptotic neurons in different treatment groups. Error bars indicate S.E.M. *P<0.05 and **P<0.01

Previous studies have suggested that increased ROS in the low magnesium model are of mitochondrial origin.^8,35^ Comonitoring ROS of mitochondrial origin and intracellular Ca^2+^ changes in neurons, using the mitochondrial ROS indicator MitoSOX and Ca^2+^ indicator Fluo-4, showed that in neurons showing low-magnesium-induced Ca^2+^ oscillations, no increase of ROS of mitochondrial origin could be detected (Figures 7a and b). We tested rates at different time points. There was a significant change in ROS production between the time points (repeat-measures ANOVA; F (2, 44)=4.1; *P*<0.05). There was a decrease in the rate of ROS generation over time that could be attributed to mitochondrial depolarization in low-magnesium-treated neurons, supporting this as a significant confounder in previous studies (Figure 7b).

### Apoptosis in the low magnesium model can be reduced by inhibiting ROS production

Excessive ROS production is known to lead to cell death.^2^ We therefore asked whether blocking sources of ROS generation might reduce cell death in the low magnesium model. The caspase-3 substrate NucView 488 allows measurements of apoptosis in real time. In time-series experiments with NucView 488, we observed apoptosis in a small fraction of neurons after ~40 min in the low magnesium conditions (Figure 7c). After 2 h, ~40% of neurons exhibited apoptosis. Pretreatment with inhibitor of NADPH oxidase assembly, gp91 ds-tat significantly reduced the proportion of neurons undergoing apoptosis in the low magnesium model of seizure-like activity (Figure 7d). To confirm this, we analysed the degree of apoptosis at 2 h in the different treatment groups. There was a significant difference in the rates of cell death between the four groups (F (3, 11)=8.43; one-way ANOVA; *P*<0.01). Adding the NADPH oxidase inhibitor gp91 ds-tat or the XO inhibitor oxypurinol to the low magnesium aCSF significantly reduced the percentage of apoptotic neurons when compared with low magnesium treatment alone (*P*<0.05; *post hoc* tests; Tukey's test; Figure 7e).

## Discussion

Using live cell imaging, we have shown that seizure-like activity induces profound changes in the rate of ROS generation in neurons. This depends on the degree of neuronal activity. ROS generation was blocked with NMDA receptor antagonists, which also abolished the low-magnesium-induced Ca^2+^ oscillations in neurons. To our surprise, low-magnesium-induced ROS generation was independent of external Ca^2+^, suggesting that a Ca^2+^-independent mechanism can trigger a cascade leading to an increase in neuronal ROS generation. Most importantly, the primary sources of ROS generation during neuronal hyperexcitability in this model were NADPH oxidase and XO, whereas mitochondrial ROS generation, which was previously suggested to be the primary source of ROS generation during seizure-like activity,^8,35^ did not contribute significantly to neuronal free radical load. Importantly, we also showed that targeting these mechanisms, by inhibiting NADPH oxidase or XO is neuroprotective.

### Low-magnesium-induced ROS generation in neurons is dependent on NMDA receptor

We found that low magnesium-induced ROS generation is dependent on repetitive epileptiform activity, as it was not seen when the activity level of neurons was low as measured with simultaneous [Ca^2+^]_c_ recordings. Previous studies have found that low-magnesium-induced Ca^2+^ oscillations and the detrimental effect of NMDA receptor activation in cultures can be abolished by blocking NMDA receptors.^29,36^ In keeping with this, we were able to abolish ROS production in the low magnesium model of seizure-like activity by NMDA receptor antagonists. In addition, we were able to block ROS production and low-magnesium-induced Ca^2+^ oscillations by selectively blocking the NR2B subunit of the NMDA receptor, suggesting that ROS production is mainly mediated by the NR2B containing NMDA receptors. This is consistent with previous evidence that indicates that NMDA receptor excitotoxicity depends on the type of subunit expressed and on the location relative to the synapse, so that the, predominantly extrasynaptic, NR2B subunit containing NMDA receptors are responsible for the detrimental effects of NMDA receptor activation on neurons.^37, 38, 39, 40^

### NADPH oxidase is the primary source of free radical generation in the low magnesium model of epilepsy and can be activated independent of Ca^2+^ flux through the NMDA receptor

Previous studies have shown that the NMDA receptor-mediated ROS increase is linked to NADPH oxidase activation.^16,17^ However, these two reports have relied on excessive pharmacologic NMDA receptor activation (40 and 100 *μ*M NMDA), raise concerns about the physiologic and pathophysiologic relevance of this finding to disease. Our study, for the first time, provides evidence and mechanistic insight into free radical generation in an *in vitro* model of an acute neurologic disease. Our results convincingly show that NMDA-receptor-dependent ROS production in neurons is dependent on NADPH oxidase activation. NADPH oxidase in neurons can be activated through Ca^2+^-mediated activation of PKC, which in turn phosphorylates p47^phox^ a subunit of the NADPH oxidase complex responsible for assembly with the NOX2 subunits.^41^ Interestingly, we found that NADPH oxidase was activated in low Mg^2+^- and Ca^2+^-free conditions, implying a mechanism of NADPH oxidase activation that is independent of Ca^2+^ entry. Our results indicate that NOX2 may rely solely on Na^+^ entry through NMDA receptors, something that has also been suggested in a study showing sodium-dependent activation of NOX2 in cardiomyocytes.^42^ Whether NADPH oxidase assembly relies on Ca^2+^-independent PKC activation in this setting remains to be determined. This finding has important therapeutic implications as it suggests that NMDA-mediated detrimental ROS generation can be maintained without excessive calcium entry. Previous reports have shown that intracellular acidification of neurons can occur during transient NMDA receptor activation.^43^ We have extended this finding by showing that activation of NMDARs during low-magnesium-induced seizure-like activity is also sufficient to induce intracellular acidification.

Several *in vivo* and *in vitro* epilepsy models support the pivotal role of NADPH oxidase in seizures and epilepsy. In keeping with our findings, an increase in NADPH oxidase activity has been linked to chemoconvulsive epilepsy models such as the pilocarpine and kainate model of epilepsy.^20,44^ Inhibition of NADPH oxidase was effective in reducing cell death in the *in vivo* pilocarpine model of temporal lobe epilepsy.^9,45^ However, our study shows for the first time that the protective effect of NADPH oxidase inhibition is independent of chemoconvulsants.

### XO contributes to a delayed increase in ROS generation in epileptiform activity

We identified that XO inhibition resulted in decreased generation of ROS during low magnesium exposure. The inhibition of ROS generation affected the late phases (>10 min) of low magnesium exposure. These findings were difficult to capture in a group analysis as the onset of the late phase varied significantly between cells, indicating that the XO-dependent secondary ROS generation is not homogeneous. Intracellular ATP depletion occurs during continuous epileptiform activity,^23,45^ and results in adenine formation and consequently an increase in hypoxanthine and xanthine, substrates for XO.^31,32^ Previous reports have shown a beneficial effect of XO inhibition on seizures.^46, 47, 48^ Our results confirm these observations, and for the first time provide evidence that inhibition of XO with oxypurinol reduces seizure-induced neuronal cell death.

### Mitochondria are not the primary site of ROS generation in the low magnesium model of epilepsy

Increased free radical generation in seizures has commonly been ascribed to mitochondrial dysfunction; this assumption has, however, often been based on the coincidence of ROS changes and changes in mitochondrial metabolism during seizures.^44,49^ Despite studies suggesting that ROS of mitochondrial origin are the major source of ROS in epilepsy and specifically in the low magnesium model of epilepsy,^8,35,50,51^ we show here that ROS of mitochondrial origin are not the primary contributors to the neuronal free radical burden. Some of the previous work has only demonstrated indirect evidence of free radical formation through measures of glutathione, a major antioxidant.^51,52^ A major confounder in the interpretation of the few studies that use HEt to measure ROS production is that it is based on the temporal relationship between the increased HEt fluorescence and Ca^2+^ transients. However, HEt can undergo fluorescent dequenching on depolarization-induced release from the mitochondrial matrix to the cytoplasm.^53^ Using MitoSOX, a more specific mitochondrial ROS indicator, we were not able to support these previous findings, and, in contrast, observed a general decrease in mitochondrial ROS generation. Mild intracellular Ca^2+^ influx stimulates respiration and consequently leads to mitochondrial membrane potential hyperpolarization. This, in turn, can slightly increase the generation of ROS.^54^ However, mitochondrial membrane potential depolarization, as occurs during prolonged seizure-like events,^14^ inhibits mitochondrial ROS generation.

**Figure 8:**
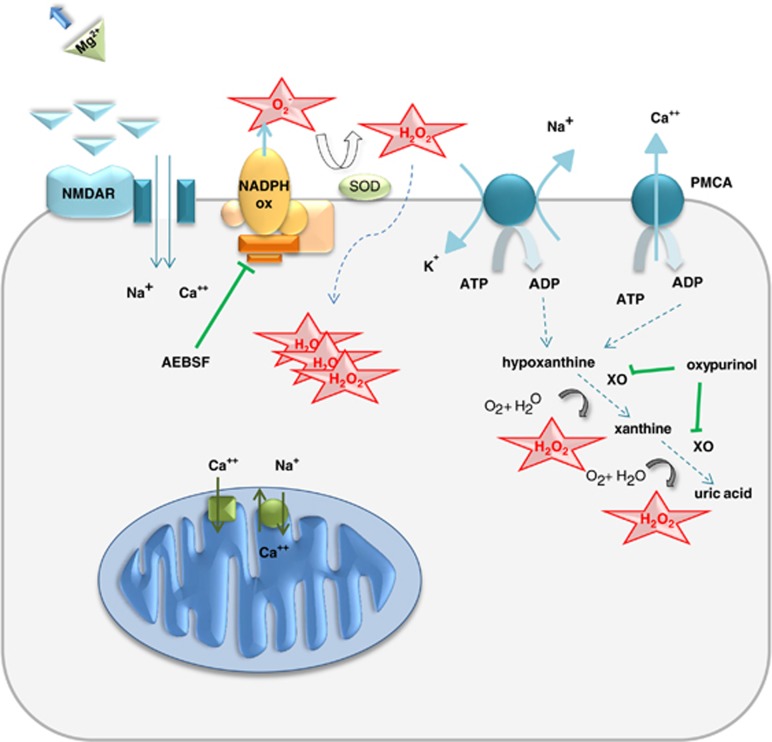
Schematic diagram illustrating the mechanisms of ROS generation in the low magnesium model of seizure-like activity: NADPH ox, NADPH oxidase; NMDAR, NMDA receptor; PMCA, plasma membrane Ca^2+^ ATPase; SOD, superoxide dismutase

For the first time, we have provided a detailed insight into the importance of different ROS-generating pathways in hyperexcitability (summary in Figure 8). Our findings of ROS generation during hyperexcitability, the time course and the sources of ROS generation with a calcium- and mitochondria-independent mechanism suggest novel interventional targets to prevent neuronal death in neurologic diseases associated with hyperexcitability, such as epilepsy, and challenge traditional views on exclusively mitochondria- and calcium-dependent excitotoxicity.

## Materials and Methods

### Cortical cell cultures

Mixed cocultures of cortical neurons and glial cells from postnatal (P0) Sprague–Dawley rats (UCL breeding colony) were prepared according to a modified protocol described by Haynes.^55^ Rat brains were quickly removed and neocortical tissue was cut and minced in ice-cold HBSS (Ca^2+^ and Mg^2+^ free; Gibco-Invitrogen, Paisley, UK). After treatment with 1% trypsin for 15 min at 37 °C to dissociate the cells, residual trypsin was removed and the tissue was triturated. The suspension was plated onto poly-D-lysine/laminin-coated coverslips and cultured in Neurobasal A medium (Gibco-Invitrogen) supplemented with B-27 and 2 mM L-glutamine (Gibco-Invitrogen). Experiments were carried out after 12–21 days *in vitro* to allow maturation of synapses in cultures. Neurons were identified and distinguished from glia by their smooth rounded somata and distinct processes using phase-contrast imaging.

### Transfection with genetically encoded pH probe HyPer-C199S

At day 11, glioneuronal cultures were transfected with plasmid coding HyPer-C199S. HyPer-C199S, a pH indicator, has been recommended as an ideal control for the genetically encoded H_2_O_2_ indicator HyPer-3, as it is the H_2_O_2_-insensitive version of the HyPer-3. Transfections were performed using Effectene transfection reagent (Qiagen, Venlo, Netherlands) yielding ~5% transfection efficiency in neurons. Cells were subjected to imaging, 1–3 days after transfection.

### Imaging of intracellular ROS generation, [Ca^2+^]_c_, ROS and [Ca^2+^]_c_ coregistration and [Na^+^]_c_

Fluorescent dyes were obtained from Invitrogen (Paisley, UK), unless otherwise stated. Preincubation and experimental procedures were performed at room temperature and all preincubations were performed in an HEPES-buffered salt solution (aCSF), composition in mM: 125 NaCl, 2.5 KCl, 2 MgCl_2_, 1.25 KH_2_PO_4_, 2 CaCl_2_, 30 glucose and 25 HEPES, pH adjusted to 7.4 with NaOH. ROS generation was measured with HEt (2 *μ*M, mostly superoxide) or H_2_DFFDA (20 *μ*M, mostly hydrogen peroxide). To avoid accumulation of oxidized products, HEt was not preincubated, but was present in solutions throughout the experiments. In experiments using H_2_DFFDA, cells were incubated for 30 min and washed before experiments. To target mitochondrial ROS production, cells were loaded with MitoSOX (10 *μ*M) for 10 min and washed before experiments. Intracellular Ca^2+^ measurements were performed using fura-2-AM (5 *μ*M) or Fluo-4 AM (5 *μ*M) and intracellular sodium levels were monitored with the ratiometric dye SBFI-AM (10 *μ*M). Ca^2+^ and Na^+^ indicators were loaded with 0.005% Pluronic F-127 for 30 min and then washed. Rates of lipid peroxidation in neurons were measured using C11-BODIPY. Glioneuronal cocultures were preincubated for 20 min with C11-BODIPY (excitation 581/emission 591; 2 *μ*M; Molecular Probes, Eugene, OR, USA). pH was monitored in neurons using carboxy-SNARF-AM (10 *μ*M), which were loaded with 0.005% Pluronic F-127 for 30 min and then washed. To correlate the rate of ROS production to Ca^2+^ changes within the cell, ROS generation and [Ca^2+^]_c_ were measured simultaneously using HEt and Fluo-4 AM. Experiments were carried out in either aCSF or excluding MgCl_2_ (low magnesium). Experiments with extracellular Ca^2+^ depletion were performed in aCSF omitting Ca^2+^ and adding the Ca^2+^ chelator EGTA (0.5 mM).

Fluorescent images were obtained on an epifluorescence inverted microscope equipped with a × 20 fluorite objective (Cairn Research, Kent, UK). Excitation wavelength was selected using a 10 nm bandpass filter centred on 340, 380, 490 or 530 nm light as appropriate. Emitted fluorescence was detected by a cooled CCD camera after passing through a long-pass filter and 12-bit resolution. Laser power, gain and black level were optimized to obtain the full dynamic range while avoiding saturation for each excitation wavelength. Fluorescence of H_2_DFFDA was excited by illumination at 490 nm, whereas HEt was excited by illumination at 530 nm. Ratiometric HEt fluorescence was recorded with excitation light at 380 and 530 nm. For most of the experiments, we chose to perform measurements of ROS production rates with HEt at a single wavelength, first to avoid photobleaching and phototoxicity from excitation of cells in the range of UV light and second based on the fact that in some experiments [Ca^2+^]_c_ was coregistered with the ROS rate measurements. It was not possible to measure H_2_DFFDA fluorescence alongside Fluo-4 fluorescence given the similar emission wavelength of these dyes. [Ca^2+^]_c_ and [Na^+^]_c_ were measured after exciting dyes with light provided by a xenon arc lamp, the beam passing through a monochromator at 340 and 380 nm with bandwidth of 10nm (Cairn Research, Kent, UK). We presented traces as the ratio of excitation at 340 and 380 nm, both with emission at >515 nm. Intracellular Na^+^ and Ca^2+^ levels were expressed as ratios and were not calibrated to avoid inaccuracies arising from different calibration methods. Phototoxicity and photobleaching of cells was minimized by limiting light exposure to the time of acquisition of the images. Fluorescent images were acquired with a frame interval of 10 s. Data were analysed using software from Andor (Belfast, UK). Illumination intensity was kept to a minimum (at 0.1–0.2% of laser output) to avoid phototoxicity and the pinhole set to give an optical slice of ~2 *μ*m. Rates of ROS increase were calculated at different time points (2 and 10 min) after exposure to low magnesium aCSF only or to low magnesium aCSF in the presence of drug. These were compared with rates recorded during a 2–5 min aCSF exposure period referred to as baseline. Experiments were repeated at least four times using more than three different cultures.

All confocal images were obtained with a Zeiss 710 LSM (Jena, Germany) with an integrated META detection system. Coregistration of ROS production and Ca^2+^ signal was performed exciting HEt with the 565 laser and measuring light emitted at 580–620 nm. Simultaneous Ca^2+^ signals were acquired, exciting Fluo-4 with the 488 nm argon laser and measuring the emitted light at 500–550 nm (x40 objective). C11-BODIPY (581/591) was excited using the 488 and 543 nm laser line and fluorescence measured using a bandpass filter from 505 to 550 nm and 560 nm long-pass filter (x40 objective). HyPer-C199S fluorescence was excited at 488 and 405 nm and emission set at 510–540 nm and expressed as 488/405 ratio. Carboxy-SNARF was excited using the 543 nm laser line and emission was collected at 580±30 and 650 ±30 nm. SNARF was expressed as 650/580 ratio and was calibrated with brief application of NH_4_Cl_2._

MitoSOX images were obtained using a x63 objective to increase precision in measuring fluorescent signals immediately over mitochondria. MitoSOX were excited using the 565 nm laser line and fluorescence measured above 580 nm. Compared with HEt, MitoSOX is less potential sensitive and undergoes significantly less fluorescent dequenching. Fluorescent dequenching indicates the release of the dye to the cytosol and highlights the importance of measuring the signal immediately over mitochondria with high magnification as in our experimental set-up.^5^

### Measuring apoptosis with NucView 488 caspase-3 substrate

Apoptosis was assessed both by measuring time series and time points within the experiment. NucView 488 caspase-3 substrate allows detection of caspase-3 activity in real time. Neuronal cultures were loaded for 15 min with 10 mM NucView 488 caspase-3 substrate (Biotium, Hayward, CA, USA). The 488 nm argon laser was used to excite NucView 488 fluorescence, which was measured using a bandpass filter from 510 and 560 nm. Using phase-contrast optics, a bright-field image allowed identification of neurons, above the glial layer.^5^

### Statistical analyses

Statistical analyses (two-tailed Student's *t*-test, one-way ANOVA, repeat-measure ANOVA, *post hoc* Tukey) were performed using SPSS 17.0 (SPSS, Chicago, IL, USA). Throughout ROS production was measured at two time points (2 and 10 min) and a repeat-measures ANOVA was used for analysis with time at the within-subject factor. The significance level was set at *P*<0.05 and all data are given as mean±S.E.M.

## Abbreviations

ROS: reactive oxygen species
XO: xanthine oxidase
PKC: protein kinase C
PMCA: plasma membrane Ca2+ ATPase
NMDAR: NMDA receptor
SOD: superoxide dismutase
HEt: hydroethidine
H2DFFDA: 5- and 6- carboxy-2',7'-dichlorofluorescein
NOX2: NADPH oxidase 2
4-AP: 4-aminopyridine

## References

[bib1] 1Abramov AY, Canevari L, Duchen MR. Beta-amyloid peptides induce mitochondrial dysfunction and oxidative stress in astrocytes and death of neurons through activation of NADPH oxidase. J Neurosci 2004; 24: 565–575. 14724257 10.1523/JNEUROSCI.4042-03.2004 PMC6729998

[bib2] 2Chinta SJ, Andersen JK. Redox imbalance in Parkinson's disease. Biochim Biophys Acta 2008; 1780: 1362–1367. 18358848 10.1016/j.bbagen.2008.02.005 PMC2547405

[bib3] 3Gandhi S, Wood-Kaczmar A, Yao Z, Plun-Favreau H, Deas E, Klupsch K et al. PINK1-associated Parkinson's disease is caused by neuronal vulnerability to calcium-induced cell death. Mol Cell 2009; 33: 627–638. 19285945 10.1016/j.molcel.2009.02.013 PMC2724101

[bib4] 4Halliwell B. Oxidative stress and neurodegeneration: where are we now? J Neurochem 2006; 97: 1634–1658. 16805774 10.1111/j.1471-4159.2006.03907.x

[bib5] 5Abramov AY, Scorziello A, Duchen MR. Three distinct mechanisms generate oxygen free radicals in neurons and contribute to cell death during anoxia and reoxygenation. J Neurosci 2007; 27: 1129–1138. 17267568 10.1523/JNEUROSCI.4468-06.2007 PMC6673180

[bib6] 6Slemmer JE, Shacka JJ, Sweeney MI, Weber JT. Antioxidants and free radical scavengers for the treatment of stroke, traumatic brain injury and aging. Curr Med Chem 2008; 15: 404–414. 18288995 10.2174/092986708783497337

[bib7] 7Frantseva MV, Velazquez JL, Hwang PA, Carlen PL. Free radical production correlates with cell death in an *in vitro* model of epilepsy. Eur J Neurosci 2000; 12: 1431–1439. 10762371 10.1046/j.1460-9568.2000.00016.x

[bib8] 8Malinska D, Kulawiak B, Kudin AP, Kovacs R, Huchzermeyer C, Kann O et al. Complex III-dependent superoxide production of brain mitochondria contributes to seizure-related ROS formation. Biochim Biophys Acta 2010; 1797: 1163–1170. 20211146 10.1016/j.bbabio.2010.03.001

[bib9] 9Pestana RRF, Kinjo ER, Hernandes MS, Britto LRG. Reactive oxygen species generated by NADPH oxidase are involved in neurodegeneration in the pilocarpine model of temporal lobe epilepsy. Neurosci Lett 2010; 484: 187–191. 20732386 10.1016/j.neulet.2010.08.049

[bib10] 10Waldbaum S, Patel M. Mitochondrial dysfunction and oxidative stress: a contributing link to acquired epilepsy?. J Bioenerg Biomembr 2010; 42: 449–455. 21132357 10.1007/s10863-010-9320-9 PMC3102435

[bib11] 11Tsai H-L, Chang C-N, Chang S-J. The effects of pilocarpine-induced status epilepticus on oxidative stress/damage in developing animals. Brain Dev 2010; 32: 25–31. 19342187 10.1016/j.braindev.2009.02.013

[bib12] 12Layton ME, Pazdernik TL. Reactive oxidant species in piriform cortex extracellular fluid during seizures induced by systemic kainic acid in rats. J Mol Neurosci 1999; 13: 63–68. 10691293 10.1385/JMN:13:1-2:63

[bib13] 13Wang X, Fang H, Huang Z, Shang W, Hou T, Cheng A et al. Imaging ROS signaling in cells and animals. J Mol Med (Berl) 2013; 91: 917–927. 23873151 10.1007/s00109-013-1067-4 PMC3730091

[bib14] 14Kovács R, Kardos J, Heinemann U, Kann O. Mitochondrial calcium ion and membrane potential transients follow the pattern of epileptiform discharges in hippocampal slice cultures. J Neurosci 2005; 25: 4260–4269. 15858052 10.1523/JNEUROSCI.4000-04.2005 PMC6725115

[bib15] 15Schauwecker PE. Neuroprotection by glutamate receptor antagonists against seizure-induced excitotoxic cell death in the aging brain. Exp Neurol 2010; 224: 207–218. 20353782 10.1016/j.expneurol.2010.03.013 PMC2885455

[bib16] 16Girouard H, Wang G, Gallo EF, Anrather J, Zhou P, Pickel VM et al. NMDA receptor activation increases free radical production through nitric oxide and NOX2. J Neurosci 2009; 29: 2545–2552. 19244529 10.1523/JNEUROSCI.0133-09.2009 PMC2669930

[bib17] 17Brennan AM, Suh SW, Won SJ, Narasimhan P, Kauppinen TM, Lee H et al. NADPH oxidase is the primary source of superoxide induced by NMDA receptor activation. Nat Neurosci 2009; 12: 857–863. 19503084 10.1038/nn.2334 PMC2746760

[bib18] 18Schiavone S, Jaquet V, Sorce S, Dubois-Dauphin M, Hultqvist M, Bäckdahl L et al. NADPH oxidase elevations in pyramidal neurons drive psychosocial stress-induced neuropathology. Transl Psychiatry 2012; 2: e111. 22832955 10.1038/tp.2012.36 PMC3365255

[bib19] 19Cristóvão AC, Guhathakurta S, Bok E, Je G, Yoo SD, Choi D-H et al. NADPH oxidase 1 mediates *α*-synucleinopathy in Parkinson's disease. J Neurosci 2012; 32: 14465–14477. 23077033 10.1523/JNEUROSCI.2246-12.2012 PMC3501265

[bib20] 20Di Maio R, Mastroberardino PG, Hu X, Montero L, Greenamyre JT. Pilocapine alters NMDA receptor expression and function in hippocampal neurons: NADPH oxidase and ERK1/2 mechanisms. Neurobiol Dis 2011; 42: 482–495. 21397025 10.1016/j.nbd.2011.02.012

[bib21] 21DeLorenzo RJ, Pal S, Sombati S. Prolonged activation of the *N*-methyl-D-aspartate receptor-Ca2+ transduction pathway causes spontaneous recurrent epileptiform discharges in hippocampal neurons in culture. Proc Natl Acad Sci USA 1998; 95: 14482–14487. 9826726 10.1073/pnas.95.24.14482 PMC24399

[bib22] 22Blair RE, Deshpande LS, Sombati S, Elphick MR, Martin BR, DeLorenzo RJ. Prolonged exposure to WIN55,212-2 causes downregulation of the CB1 receptor and the development of tolerance to its anticonvulsant effects in the hippocampal neuronal culture model of acquired epilepsy. Neuropharmacology 2009; 57: 208–218. 19540252 10.1016/j.neuropharm.2009.06.007 PMC2757117

[bib23] 23Kovac S, Domijan A-M, Walker MC, Abramov AY. Prolonged seizure activity impairs mitochondrial bioenergetics and induces cell death. J Cell Sci. 2012; 125(Part 7): 1796–1806. 22328526 10.1242/jcs.099176 PMC4195235

[bib24] 24Kovacs R, Kardos J, Heinemann U, Kann O. Mitochondrial calcium ion and membrane potential transients follow the pattern of epileptiform discharges in hippocampal slice cultures. J Neurosci 2005; 25: 4260–4269. 15858052 10.1523/JNEUROSCI.4000-04.2005 PMC6725115

[bib25] 25Kovacs R, Schuchmann S, Gabriel S, Kardos J, Heinemann U. Ca^2+^ signalling and changes of mitochondrial function during low-Mg^2+^-induced epileptiform activity in organotypic hippocampal slice cultures. Eur J Neurosci 2001; 13: 1311–1319. 11298791 10.1046/j.0953-816x.2001.01505.x

[bib26] 26Barbarosie M, Avoli M. CA3-driven hippocampal–entorhinal loop controls rather than sustains *in vitro* limbic seizures. J Neurosci 1997; 17: 9308–9314. 9364076 10.1523/JNEUROSCI.17-23-09308.1997 PMC6573610

[bib27] 27Barkai E, Friedman A, Grossman Y, Gutnick MJ. Laminar pattern of synaptic inhibition during convulsive activity induced by 4-aminopyridine in neocortical slices. J Neurophysiol 1995; 73: 1462–1467. 7643161 10.1152/jn.1995.73.4.1462

[bib28] 28Sun DA, Sombati S, DeLorenzo RJ. Glutamate injury-induced epileptogenesis in hippocampal neurons: an *in vitro* model of stroke-induced ‘epilepsy'. Stroke 2001; 32: 2344–2350. 11588324 10.1161/hs1001.097242

[bib29] 29DeLorenzo RJ, Sun DA, Deshpande LS. Cellular mechanisms underlying acquired epilepsy: the calcium hypothesis of the induction and maintainance of epilepsy. Pharmacol Ther 2005; 105: 229. 15737406 10.1016/j.pharmthera.2004.10.004 PMC2819430

[bib30] 30Diatchuk V, Lotan O, Koshkin V, Wikstroem P, Pick E. Inhibition of NADPH oxidase activation by 4-(2-aminoethyl)-benzenesulfonyl fluoride and related compounds. J Biol Chem 1997; 272: 13292–13301. 9148950 10.1074/jbc.272.20.13292

[bib31] 31Kinugasa Y, Ogino K, Furuse Y, Shiomi T, Tsutsui H, Yamamoto T et al. Allopurinol improves cardiac dysfunction after ischemia–reperfusion *via* reduction of oxidative stress in isolated perfused rat hearts. Circ J 2003; 67: 781–787. 12939555 10.1253/circj.67.781

[bib32] 32Xia Y, Zweier JL. Substrate control of free radical generation from xanthine oxidase in the postischemic heart. J Biol Chem 1995; 270: 18797–18803. 7642530 10.1074/jbc.270.32.18797

[bib33] 33Rey FE, Cifuentes ME, Kiarash A, Quinn MT, Pagano PJ. Novel competitive inhibitor of NAD(P)H oxidase assembly attenuates vascular O(2)(−) and systolic blood pressure in mice. Circ Res 2001; 89: 408–414. 11532901 10.1161/hh1701.096037

[bib34] 34Elnakish MT, Hassanain HH, Janssen PM, Angelos MG, Khan M. Emerging role of oxidative stress in metabolic syndrome and cardiovascular diseases: important role of Rac/NADPH oxidase. J Pathol 2013; 231: 290–300. 24037780 10.1002/path.4255

[bib35] 35Kovács R, Schuchmann S, Gabriel S, Kann O, Kardos J, Heinemann U. Free radical-mediated cell damage after experimental status epilepticus in hippocampal slice cultures. J Neurophysiol 2002; 88: 2909–2918. 12466417 10.1152/jn.00149.2002

[bib36] 36Mangan PS, Kapur J. Factors underlying bursting behavior in a network of cultured hippocampal neurons exposed to zero magnesium. J Neurophysiol 2004; 91: 946–957. 14534286 10.1152/jn.00547.2003 PMC2892720

[bib37] 37Hardingham GE, Fukunaga Y, Bading H. Extrasynaptic NMDARs oppose synaptic NMDARs by triggering CREB shut-off and cell death pathways. Nat Neurosci 2002; 5: 405–414. 11953750 10.1038/nn835

[bib38] 38Liu F-Y, Wang X-F, Li M-W, Li J-M, Xi Z-Q, Luan G-M et al. Upregulated expression of postsynaptic density-93 and *N*-methyl-D-aspartate receptors subunits 2B mRNA in temporal lobe tissue of epilepsy. Biochem Biophys Res Commun 2007; 358: 825–830. 17506987 10.1016/j.bbrc.2007.05.010

[bib39] 39Monyer H, Burnashev N, Laurie DJ, Sakmann B, Seeburg PH. Developmental and regional expression in the rat brain and functional properties of four NMDA receptors. Neuron 1994; 12: 529–540. 7512349 10.1016/0896-6273(94)90210-0

[bib40] 40Tovar KR, Westbrook GL. The incorporation of NMDA receptors with a distinct subunit composition at nascent hippocampal synapses *in vitro*. J Neurosci 1999; 19: 4180–4188. 10234045 10.1523/JNEUROSCI.19-10-04180.1999 PMC6782704

[bib41] 41Bedard K, Krause K-H. The NOX family of ROS-generating NADPH oxidases: physiology and pathophysiology. Physiol Rev 2007; 87: 245–313. 17237347 10.1152/physrev.00044.2005

[bib42] 42Balteau M, Tajeddine N, de Meester C, Ginion A, Des Rosiers C, Brady NR et al. NADPH oxidase activation by hyperglycaemia in cardiomyocytes is independent of glucose metabolism but requires SGLT1. Cardiovasc Res 2011; 92: 237–246. 21859816 10.1093/cvr/cvr230

[bib43] 43Rathje M, Fang H, Bachman JL, Anggono V, Gether U, Huganir RL et al. AMPA receptor pHluorin-GluA2 reports NMDA receptor-induced intracellular acidification in hippocampal neurons. Proc Natl Acad Sci USA 2013; 110: 14426–14431. 23940334 10.1073/pnas.1312982110 PMC3761605

[bib44] 44Patel M, Li Q-Y, Chang L-Y, Crapo J, Liang L-P. Activation of NADPH oxidase and extracellular superoxide production in seizure-induced hippocampal damage. J Neurochem 2005; 92: 123–131. 15606902 10.1111/j.1471-4159.2004.02838.x

[bib45] 45Kim JH, Jang BG, Choi BY, Kim HS, Sohn M, Chung TN et al. Post-treatment of an NADPH oxidase inhibitor prevents seizure-induced neuronal death. Brain Res 2013; 1499: 163–172. 23313582 10.1016/j.brainres.2013.01.007

[bib46] 46Kramer LD, Locke GE, Nelson LG, Ogunyemi AO. Status epilepticus following withdrawal of allopurinol. Ann Neurol 1990; 27: 691. 10.1002/ana.410270620 2360809

[bib47] 47Tada H, Morooka K, Arimoto K, Matsuo T. Clinical effects of allopurinol on intractable epilepsy. Epilepsia 1991; 32: 279–283. 1900791 10.1111/j.1528-1157.1991.tb05256.x

[bib48] 48Zagnoni PG, Bianchi A, Zolo P, Canger R, Cornaggia C, D'Alessandro P et al. Allopurinol as add-on therapy in refractory epilepsy: a double-blind placebo-controlled randomized study. Epilepsia 1994; 35: 107–112. 8112231 10.1111/j.1528-1157.1994.tb02919.x

[bib49] 49Cock H. The role of mitochondria in status epilepticus. Epilepsia 2007; 48: 24–27. 18329991 10.1111/j.1528-1167.2007.01341.x

[bib50] 50Liang L-P, Patel M. Mitochondrial oxidative stress and increased seizure susceptibility in Sod2(−/+) mice. Free Radic Biol Med 2004; 36(Suppl 8): 542–554. 14980699 10.1016/j.freeradbiomed.2003.11.029

[bib51] 51Liang L-P, Patel M. Seizure-induced changes in mitochondrial redox status. Free Radic Biol Med 2006; 40: 316–322. 16413413 10.1016/j.freeradbiomed.2005.08.026

[bib52] 52Cock HR, Tong X, Hargreaves IP, Heales SJR, Clark JB, Patsalos PN et al. Mitochondrial dysfunction associated with neuronal death following status epilepticus in rat. Epilepsy Res 2002; 48: 157–168. 11904234 10.1016/s0920-1211(01)00334-5

[bib53] 53Budd SL, Castilho RF, Nicholls DG. Mitochondrial membrane potential and hydroethidine-monitored superoxide generation in cultured cerebellar granule cells. FEBS Lett 1997; 415: 21–24. 9326361 10.1016/s0014-5793(97)01088-0

[bib54] 54Nicholls DG. Mitochondria and calcium signaling. Cell Calcium 2005; 38: 311–317. 16087232 10.1016/j.ceca.2005.06.011

[bib55] 55Haynes LW. The Neuron in Tissue Culture. New Yok, NY, USA: Wiley, 1999.

