# Supplementary figures and images for: Seizure activity results in calcium- and mitochondria-independent ROS production via NADPH and xanthine oxidase activation
Source: Cell Death Dis. 2014 Oct 2;5(10):e1442–. doi: 10.1038/cddis.2014.390 (PMC4649505; doi:10.1038/cddis.2014.390)

**A**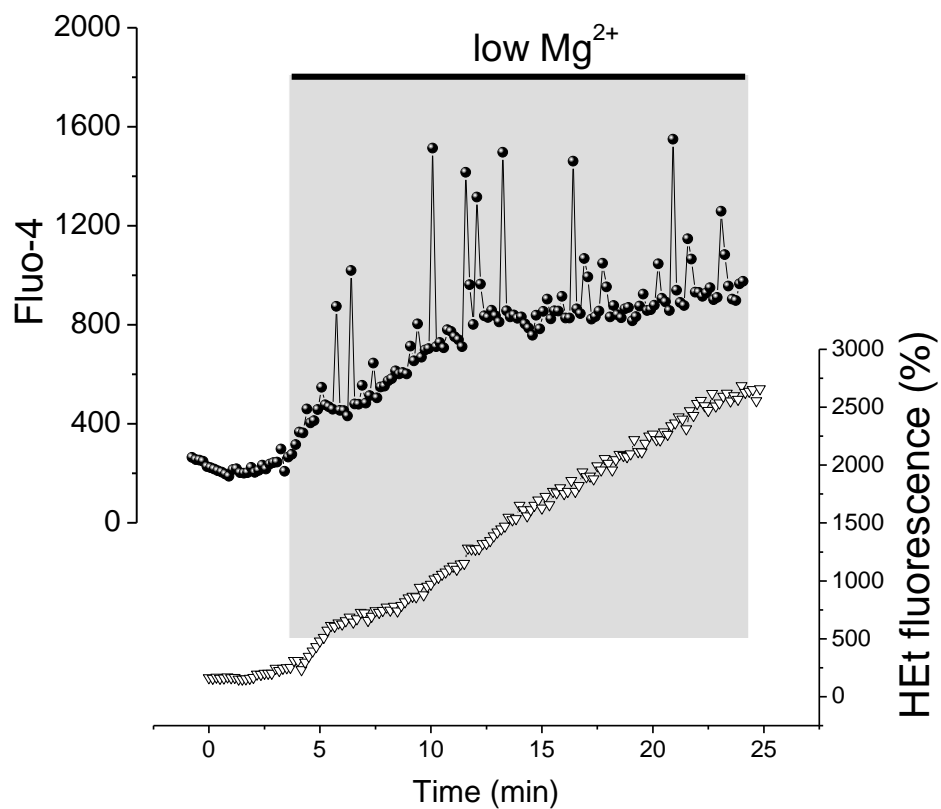**B**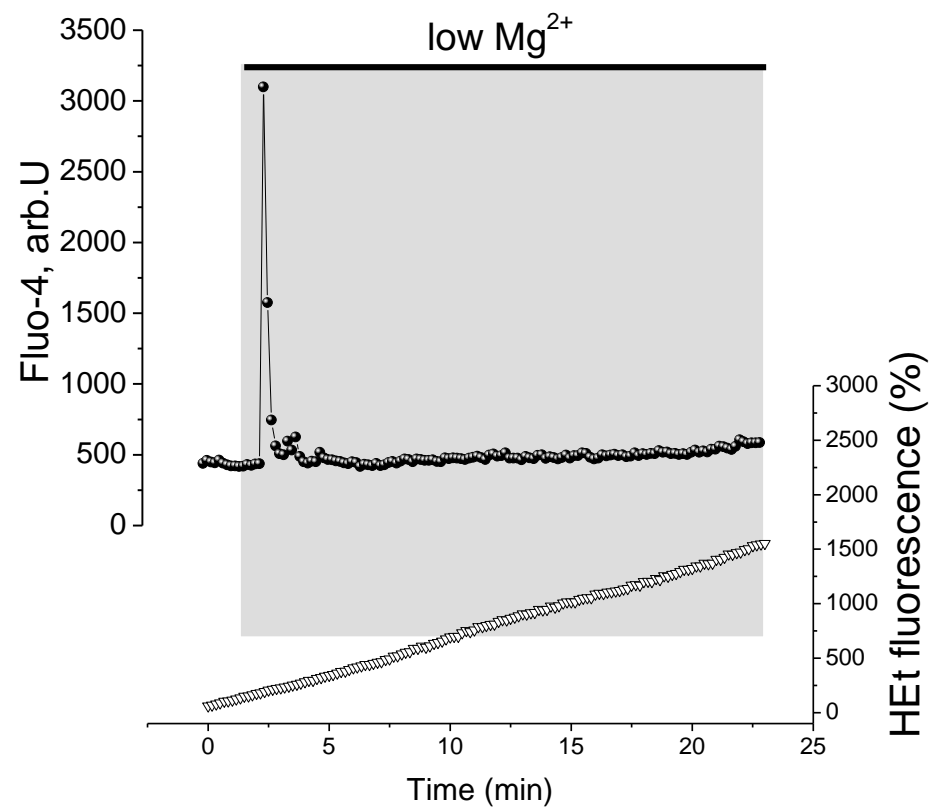

Supplement: Supplementary Figure 1 [file cddis2014390x2.pdf]

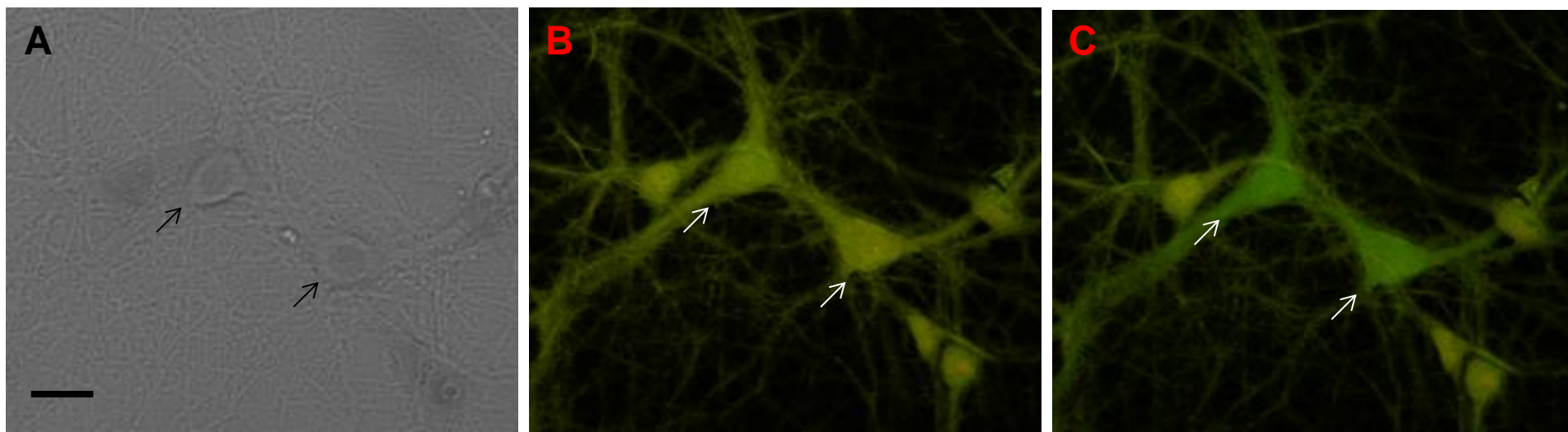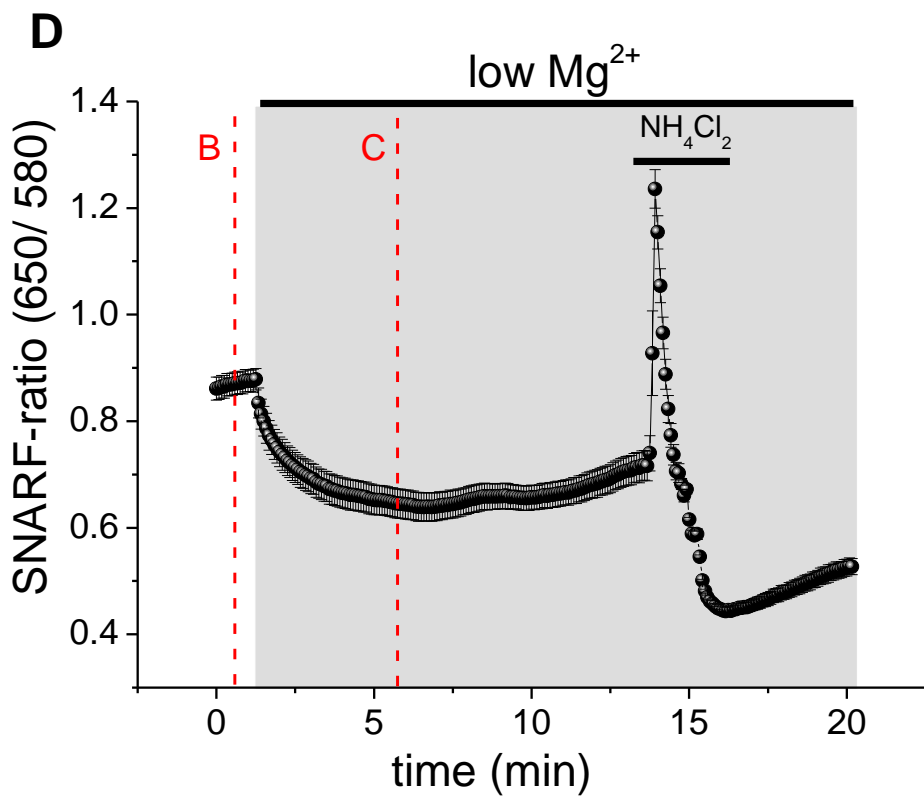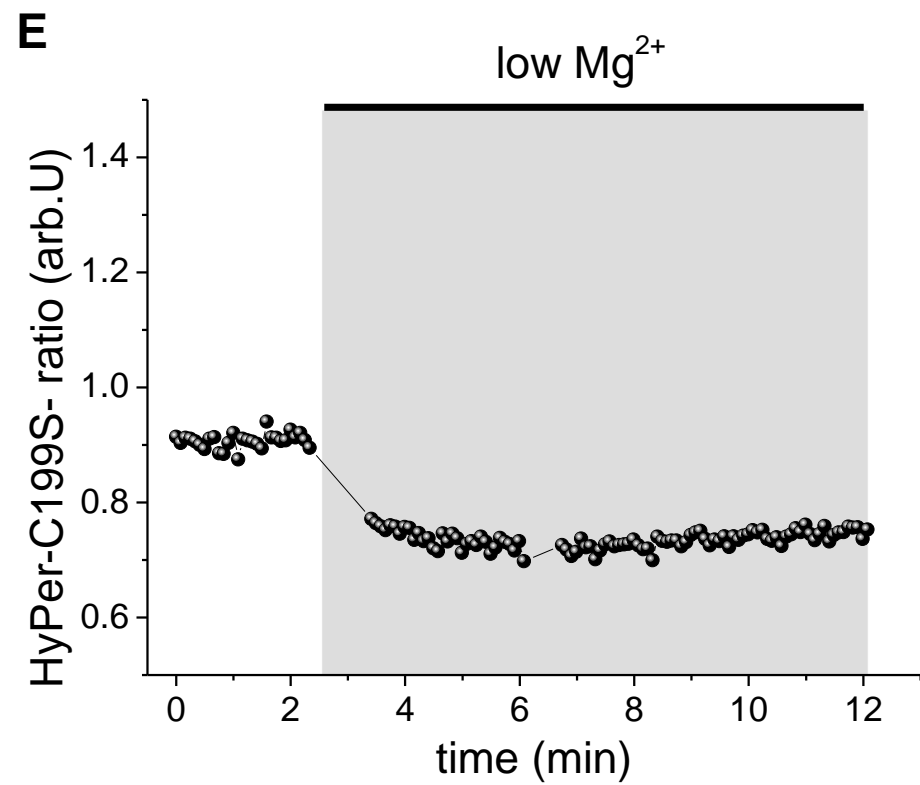

**Suppl. Fig. 2**

Supplement: Supplementary Figure 2 [file cddis2014390x3.pdf]

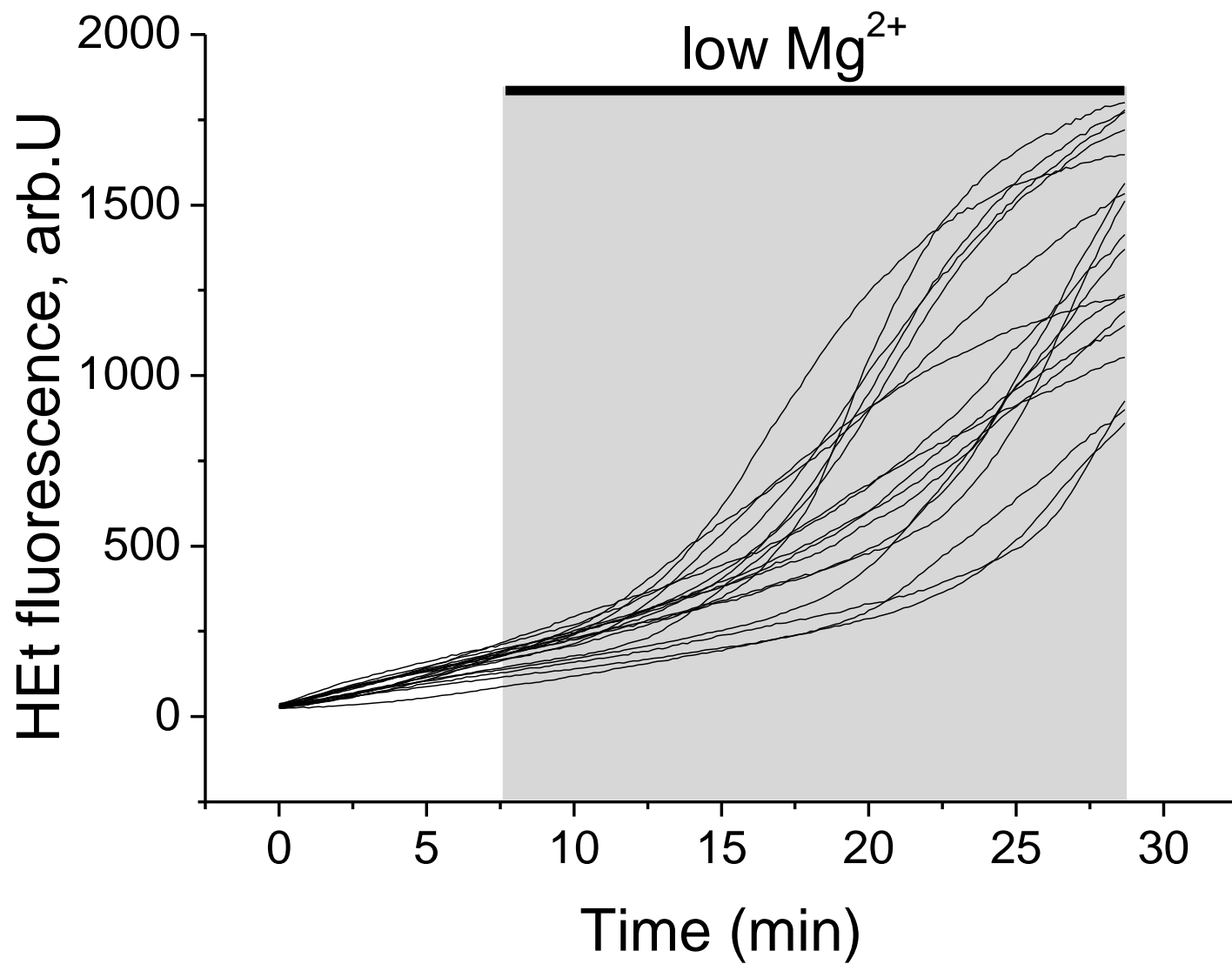

**Suppl. Fig. 3**

Supplement: Supplementary Figure 3 [file cddis2014390x4.pdf]

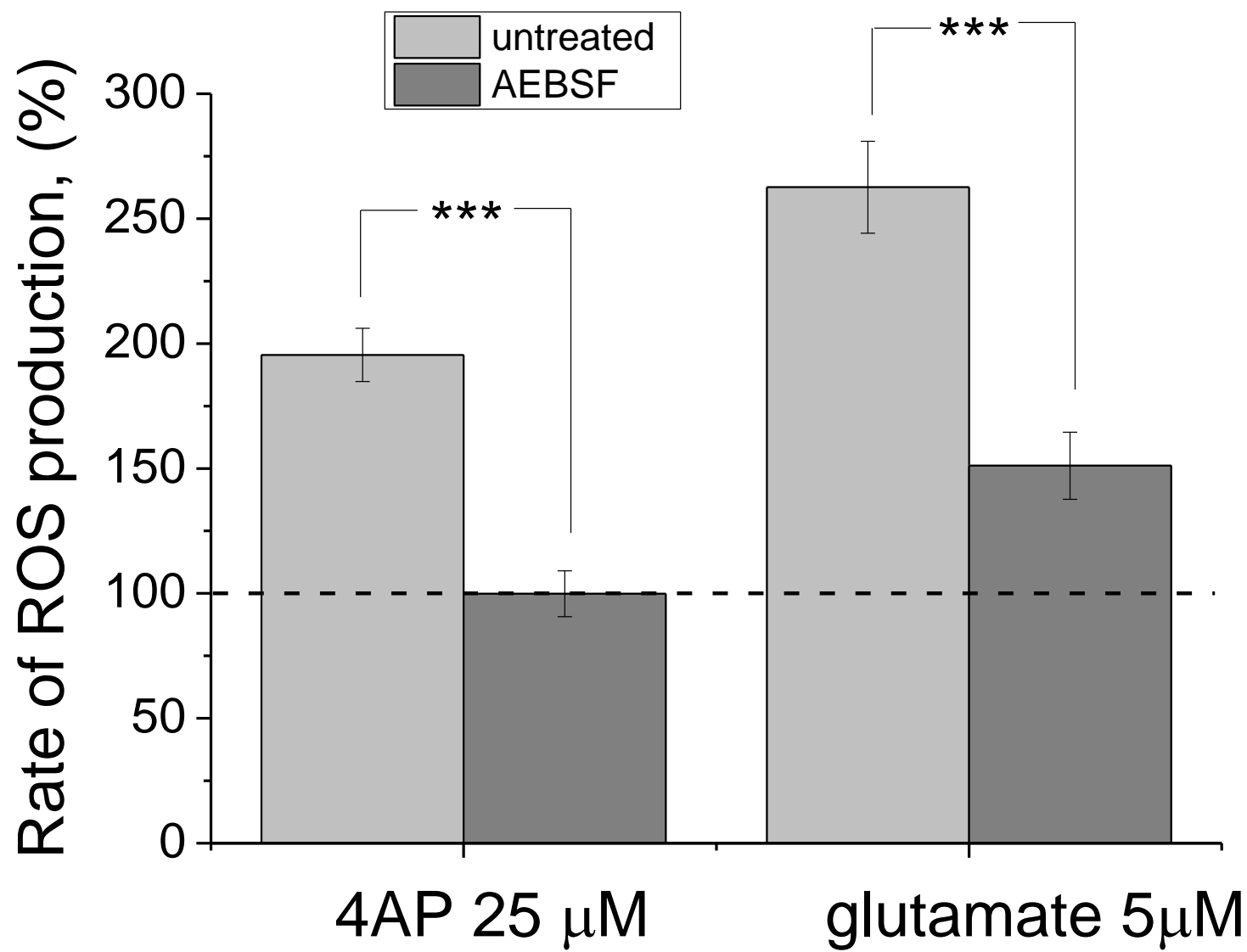

Suppl. Fig. 4

Supplement: Supplementary Figure 4 [file cddis2014390x5.pdf]
