# Supplementary material for: Seizure activity results in calcium- and mitochondria-independent ROS production via NADPH and xanthine oxidase activation
Source: Cell Death Dis. 2014 Oct 2;5(10):e1442–. doi: 10.1038/cddis.2014.390 (PMC4649505; doi:10.1038/cddis.2014.390)
Supplement: Supplementary Video and Figure Legend [file cddis2014390x6.doc]

**Suppl. Video 1 Legend**

Seizure-like activity in neurons induced by the low magnesium medium, as indicated by repetitive Ca^2+^ oscillations measured with the calcium indicator fluo-4.

**Suppl. Figure Legends**

Suppl. Fig. 1: *ROS changes in the in vitro low magnesium model are activity dependent:* (A+B) Calcium (Fluo-4; upper panel) and ROS (HEt; lower panel) co-registration in single, representative neurons. (A) The neuron representative of neurons that exhibit frequent low-magnesium-induced Ca^2+^ oscillations indicative of a high epileptiform activity whereas (B) the trace representative of a neuron showing a low degree of epileptiform activity.

Suppl. Fig. 2: *pH changes in the low magnesium model of seizure-like activity*: (A-D) Low magnesium treatment induces acidification in neurons as measured with SNARF. (A) Phase-contrast image of neurons in culture. The black arrow points at two neuronal bodies which appear smooth rounded. (B) Carboxy SNARF ratio image of the same region showing (580/650) emission ratio under baseline conditions. (C) Treatment with low magnesium induces intracellular acidification in neurons, but not in astrocytes. (D) Time-course of the pH changes in a representative experiment (mean ± SEM) expressed as Carboxy SNARF ratio. (E) The trace of a single neuron showing alterations in Hyper C199S-ratio concordant with acidification of the cell during low magnesium seizure-like activity.

Suppl. Fig.3: *Differences in the onset of the secondary ROS increase between neurons:* Single traces represent the HEt signal in neurons as recorded during low magnesium exposure. Note that the onset of the secondary ROS increase as represented by the steep slope differs substantially between single neurons.

Suppl. Fig. 4: *ROS changes in neurons during 4-aminopyridine (4-AP) and glutamate induced epileptiform activity can be blocked by NADPH oxidase inhibition with AEBSF:* Histogram summarizing the effect of AEBSF on 4-AP and glutamate induced ROS production. Error bars indicate SEM. *** p<0.001.
